# Predictive Features of Malignancy in Branch Duct Type Intraductal Papillary Mucinous Neoplasm of the Pancreas: A Meta-Analysis

**DOI:** 10.3390/cancers12092618

**Published:** 2020-09-14

**Authors:** Wooil Kwon, Youngmin Han, Yoonhyeong Byun, Jae Seung Kang, Yoo Jin Choi, Hongbeom Kim, Jin-Young Jang

**Affiliations:** Department of Surgery and Cancer Research Institute, Seoul National University College of Medicine, Seoul 03080, Korea; willdoc@snu.ac.kr (W.K.); views@snu.ac.kr (Y.H.); yoonhyeong@snu.ac.kr (Y.B.); 74398@snuh.org (J.S.K.); 74401@snuh.org (Y.J.C.); surgeonkhb@snu.ac.kr (H.K.)

**Keywords:** branch duct intraductal papillary mucinous neoplasm, risk factor, malignancy, meta-analysis

## Abstract

**Simple Summary:**

Currently, there are several guidelines that are widely used to establish the treatment strategy for branch duct type intraductal papillary mucinous neoplasms. Although there are some common grounds, there are discrepancies on which features they adopt, how much each feature is weighted, and how the features are combined. Furthermore, some of the features are based on lower level evidences or expert opinions. The aim of this meta-analysis was to investigate important clinical, radiological, and biochemical risk factors for malignancy and their impact as predictors. This study found symptom, size, cyst wall thickening, presence of mural nodule, change in main pancreatic duct caliber, lymphadenopathy, CA 19-9, and CEA as risk factors. Lymphadenopathy (odd ratio [OR]: 8.55), abrupt caliber change (OR: 7.41), and mural nodule (OR: 4.10) had the highest odd ratios. We expect the higher level evidences of this study to help shape better guidelines and reduce discrepancies among future guidelines.

**Abstract:**

The current guidelines on branch duct type intraductal papillary mucinous neoplasm (BD-IPMN) recommend various predictive features of malignancy as well as different treatment strategies. This study aimed to identify the risk factors for malignancy with higher level of evidence. A meta-analysis was performed on 40 literatures published between 2000 and 2019. These literatures included 6301 patients with pathologically proven IPMN. Malignancy was defined as high-grade dysplasia and invasive carcinoma. It was significantly associated with symptoms (odds ratio [OR] 1.35, confidence interval [CI] 1.01–1.79), size ≥ 3 cm (OR 1.90, CI 1.51–2.40), cystic wall thickening (OR 2.53, CI 1.50–4.27), mural nodule (OR 4.10, CI 3.38–4.97), main pancreatic duct dilatation (OR 2.98, CI 2.11–4.21), abrupt caliber change of the pancreatic duct (OR 7.41, CI 2.49–22.06), lymphadenopathy (OR 8.55, CI 3.25–22.51), elevated carbohydrate antigen 19-9 (OR 4.01, CI 2.55–6.28), and elevated carcinoembryonic antigen (OR 2.04, CI 1.60–2.61). Multilocular cysts and multiple cysts did not show a significant association with malignancy. This study examined the clinical, radiological, and biochemical features of BD-IPMN, often used as malignancy predictors according to the widely used guidelines. The results confirmed that all the features currently being used are valid.

## 1. Introduction

Branch duct type intraductal papillary mucinous neoplasm (BD-IPMN) is a well-known premalignant lesion of the pancreas. The prevalence of BD-IPMN-associated malignancy is reportedly over 24% [1]. Nearly four decades have passed since the first report of IPMN by Ohashi et al. [2], but our understanding of IPMN is still limited. Particularly, the ability to predict malignancy and set an appropriate treatment plan is far from satisfactory. Given that pancreatic cancer is the fourth leading cause of cancer mortality [3], the clinical implications of this shortcoming are grave. To make matters worse, the incidence of IPMN is on a steady rise, as incidental detections are increasing due to better access to heath check-ups and increased use of cross-sectional imaging studies [4,5]. The current situation poses a great challenge for pancreatic surgeons and physicians.

Many investigations have been conducted, results have been produced, and the endeavor continues. Currently, there are several management guidelines for IPMN. Among them, the most frequently referenced are those by the American Gastroenterological Association (AGA) [6], European Study Group on Cystic Tumours of the Pancreas [7,8], and International Association of Pancreatology (IAP) [1,9,10]. Although all these guidelines have some commonalities, they do differ with respect to certain surgical treatment indications and surveillance strategies. Another issue is that few of these guidelines cite studies with lower levels of evidences, while others cite experts’ opinions.

The first step in producing high-quality treatment guidelines for BD-IPMN is to clarify the risk factors for malignancy. Therefore, a meta-analysis was performed to identify the clinically important risk factors for malignancy and their impact. This study investigated the comprehensive factors including clinical, radiological, and biochemical factors that could be acquired preoperatively.

## 2. Results

### 2.1. Search Results

**Figure 1:**
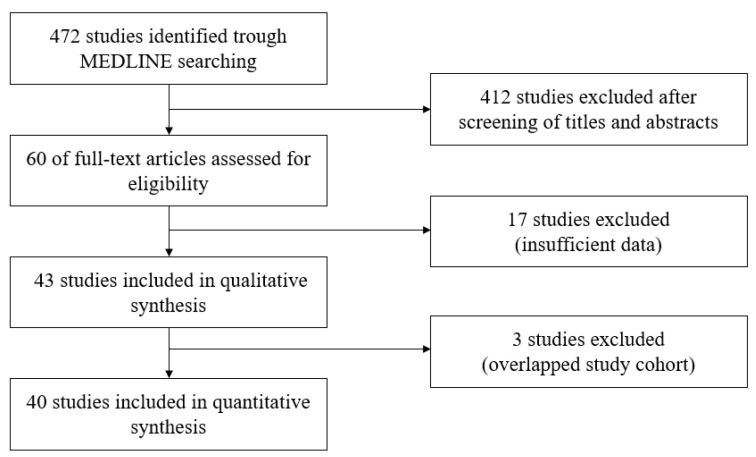
A flow diagram of the inclusion criteria of studies eligible for meta-analysis.

The search process is described in Figure 1. A thorough literature search on MEDLINE identified 472 publications that were potentially relevant to this study. A total of, 412 studies were excluded after screening. Of the remaining 60 publications, 17 were excluded after detailed review due to insufficient data regarding worrisome features/high-risk stigmata, absence of pathological data, insufficient sample size, or overlap with another study. When an overlapping study cohort was found, the larger sample study was chosen. If there were results regarding worrisome features/high-risk stigmata in a smaller overlapping study that was not addressed in the larger one, it was still included. Finally, 40 publications were included in the analysis [11,12,13,14,15,16,17,18,19,20,21,22,23,24,25,26,27,28,29,30,31,32,33,34,35,36,37,38,39,40,41,42,43,44,45,46,47,48,49,50]. The publication bias was assessed visually by inspecting the funnel plot for asymmetry. 

### 2.2. Characteristics of Included Studies

The characteristics of the included publications are described in Table 1. This study included 40 publications with 6301 patients diagnosed with IPMN, of which histological data of 4241 patients diagnosed with benign IPMN and 2060 with malignant IPMN were identified. In all studies, malignant IPMN was defined as invasive carcinoma and high-grade dysplasia. Terms such as invasive cancer, intraductal papillary mucinous carcinoma (IPMC), and invasive IPMN were considered equivalent to invasive carcinoma. Non-invasive carcinoma, carcinoma in situ, and IPMC in situ were considered equivalent to high-grade dysplasia.

### 2.3. Clinical Symptoms

**Figure 2:**
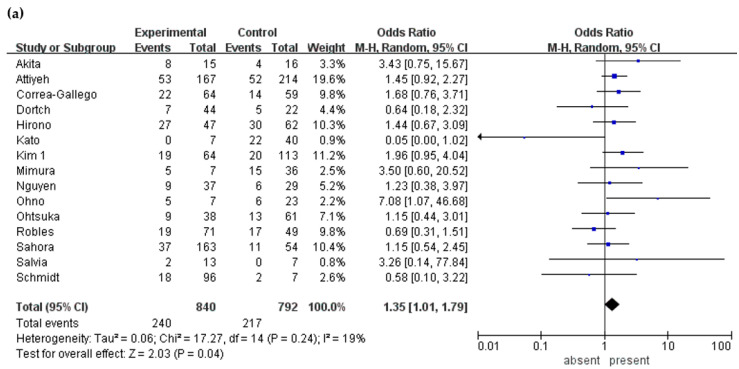

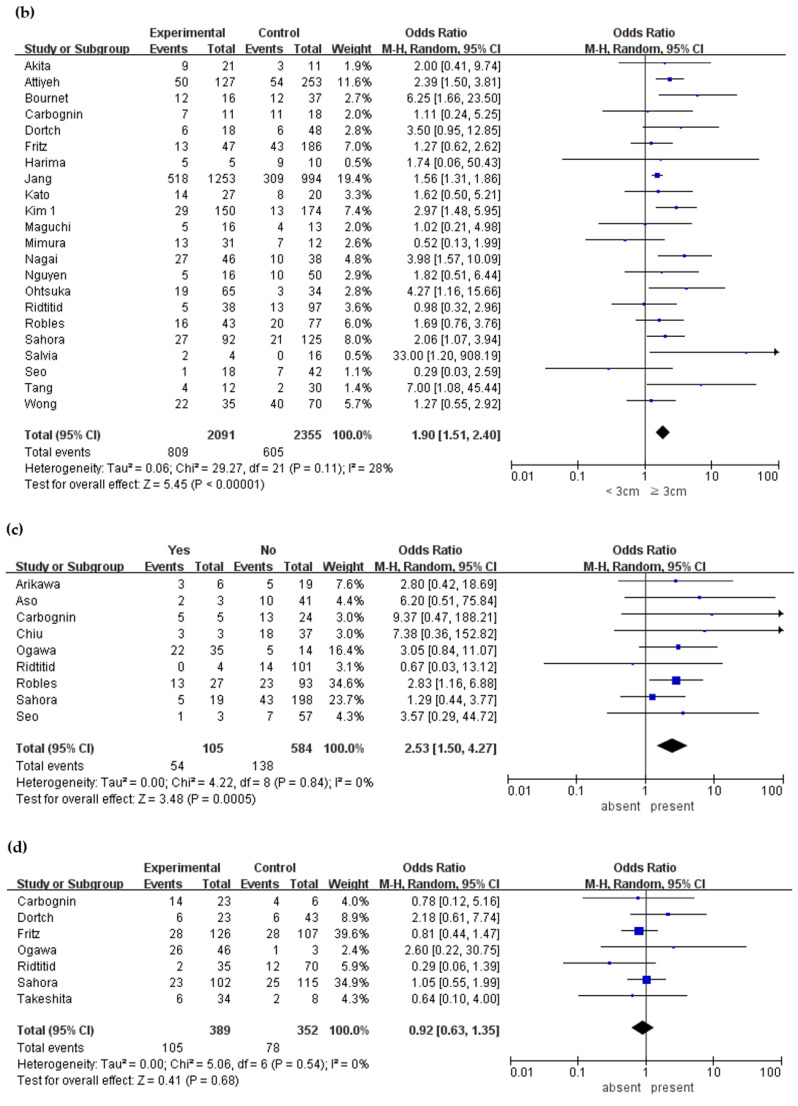

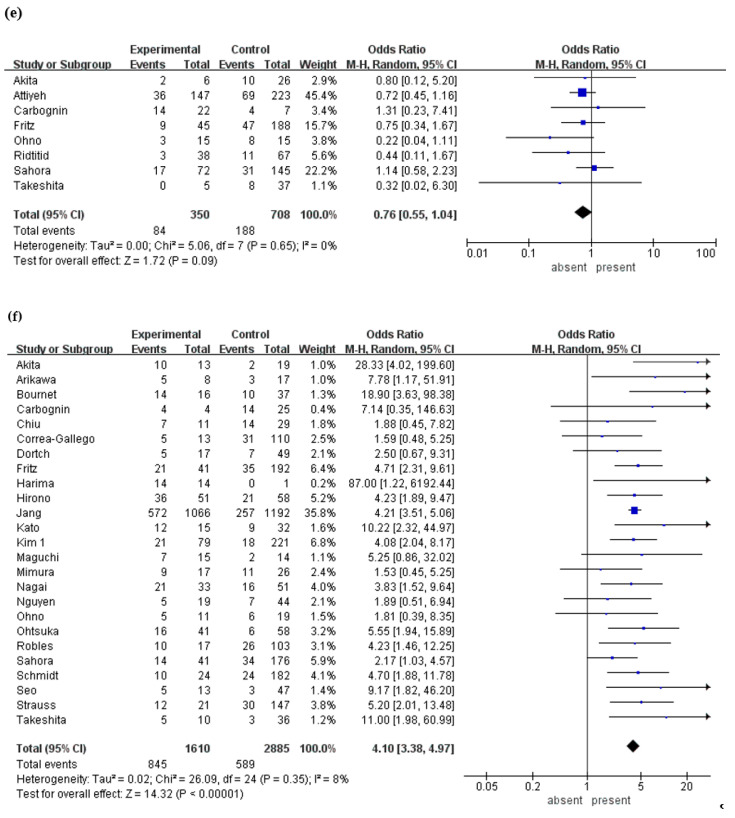
Forest plot showing the incidence of malignancy in BD-IPMN stratified by symptoms, characteristics of the cyst, and presence of mural nodule. (**a**) symptom. (**b**) cyst size. (**c**) cyst wall thickening. (**d**) multilocular cyst. (**e**) multiple cyst. and (**f**) mural nodule.

Data regarding symptoms were extractable in 15 studies [12,15,19,20,23,25,27,31,33,35,36,38,40,41,42]. In these studies, 840 patients (54.8%) presented symptoms, and malignancy was reported in 28.6% of patients with symptoms and 27.4% without symptoms. The odds ratio (OR) of having symptoms was 1.35 (95% confidence interval [CI] 1.01–1.79, *p* = 0.040) (Table 2, Figure 2a).

### 2.4. Characteristics of Cyst

Data regarding cyst size were obtained from 22 studies with 4446 patients [12,15,16,17,20,21,22,24,25,26,30,31,32,33,36,37,38,40,41,43,48,49], and the risk of malignancy was examined for a reference size of 3 cm. The malignancy rate in cysts ≥ 3 cm and < 3 cm in size was 38.7% and 25.7%, respectively. Cysts of size ≥ 3 cm significantly increased the risk of malignancy with an OR of 1.90 (95% CI 1.51–2.40, *p* < 0.001) (Table 2, Figure 2b).

Data regarding cystic wall thickening was extracted from nine studies with 689 patients [13,14,17,18,34,37,38,40,43], and wall thickening was found in 15.2% of the cases. Moreover, 51.4% of the patients with wall thickening reported malignancy as compared to 23.6% of those without wall thickening. Wall thickening was significantly associated with malignancy with OR of 2.53 (95% CI 1.50–4.27, *p* < 0.001) (Table 2, Figure 2c).

Multilocularity and multiplicity was analyzed in seven [17,20,21,34,37,40,47] and eight studies [12,15,17,21,35,37,40,47], respectively. Malignancy rate of multilocular and unilocular cysts was 27.0% and 22.2%, respectively. Furthermore, the malignancy rate of single and multiple cysts was 26.6% and 24.0%, respectively. Notably, neither of the features was associated with an increased risk of malignancy (multilocularity: OR 0.92, 95% CI 0.63–1.35, *p* = 0.680; multiplicity: OR 0.76, 95% CI 0.55–1.04, *p* = 0.090) (Table 2, Figure 2d,e)

### 2.5. Mural Nodule

Mural nodule was the most frequently investigated parameter observed in 25 studies and cohort of 4495 patients [12,13,16,17,18,19,20,21,22,23,24,25,26,30,31,32,33,35,36,38,40,42,43,46,47]. The prevalence of mural nodule in BD-IPMN was 35.8%, and the pooled malignancy rate was 31.9%. The malignancy rate was 52.5% in the presence of mural nodule and 20.4% in its absence. The presence of mural nodule resulted in a four-fold increase in the malignancy risk. The pooled OR was 4.10 (95% CI 3.38–4.97, *p* < 0.001) (Table 2, Figure 2f).

### 2.6. Changes in Main Pancreatic Duct

**Figure 3:**
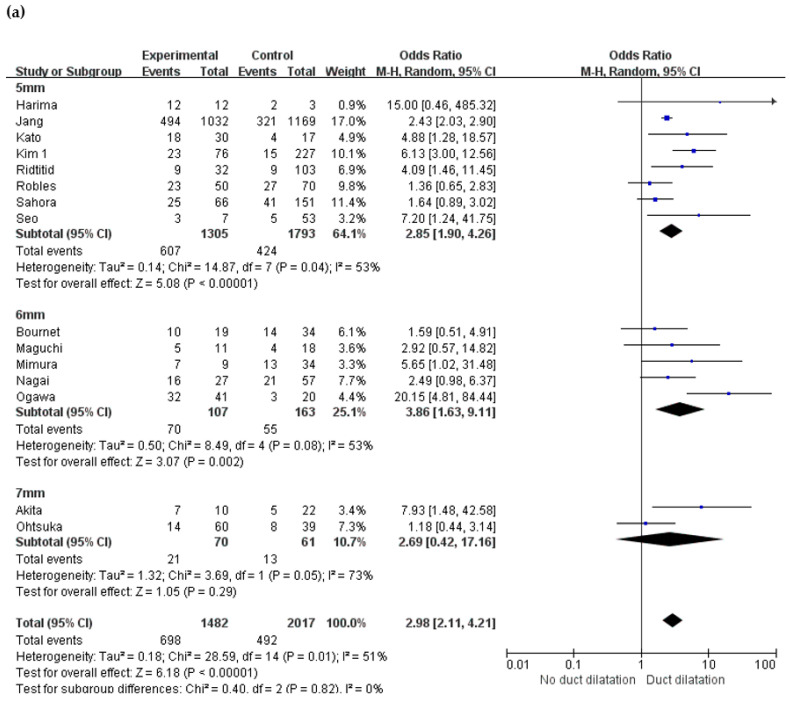

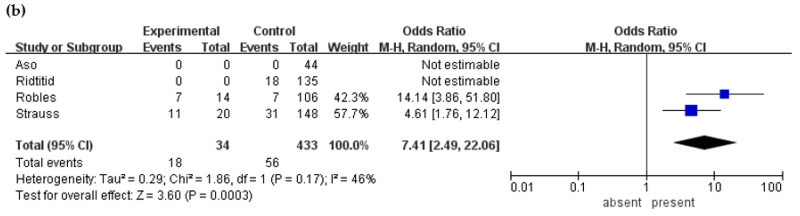
Forest plot demonstrates the incidence of malignancy in BD-IPMN in relation to change in main pancreatic duct. Forest plot stratified (**a**) by the diameter of pancreatic duct and (**b**) by abrupt caliber change.

Several studies examined the size of the main pancreatic duct, but they all had different cut-off values. The reference size was 5 mm in eight studies [22,24,25,26,37,38,40,43], 6 mm in five studies [16,30,31,32,34], and 7 mm in two studies [12,36]. For pancreatic ducts of size 5 mm, the OR was 2.85 (95% CI 1.90–4.26, *p* < 0.001), and a malignancy rate of 46.5% for ducts > 5 mm. The ORs for main pancreatic ducts > 6 and 7 mm were 3.86 (95% CI 1.63–9.11, *p* = 0.002) and 2.69 (95% CI 0.42–17.16, *p* = 0.29), respectively. Overall, the OR for dilatation of the main pancreatic duct was 2.98 (95% CI 2.11–4.21, *p* < 0.001) (Table 2, Figure 3a).

Four studies examined the caliber change in the pancreatic duct in 467 patients [14,37,38,46]. Among 34 patients with an abrupt change in caliber, 18 patients (52.9%) had malignant BD-IPMN with OR of 7.41 (95% CI 2.49–22.06, *p* < 0.001) (Table 2, Figure 3b).

### 2.7. Lymphadenopathy

**Figure 4:**
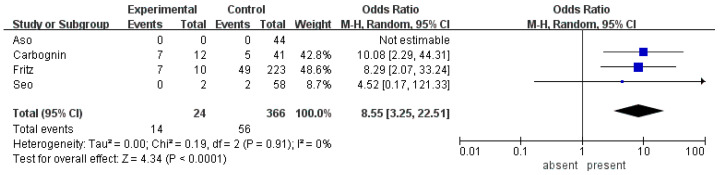
Forest plot demonstrates the incidence of malignancy in BD-IPMN stratified by the presence of lymphadenopathy.

Four studies examining lymphadenopathy had a pooled cohort of 390 patients [14,17,21,43]. The prevalence of lymphadenopathy was 6.2%. The malignancy rate in these patients was 58.3% as compared to 15.3% in those without lymphadenopathy. The OR for lymphadenopathy was the highest among all parameters at 8.55 (95% CI 3.25–22.51, *p* < 0.001) (Table 2, Figure 4)

### 2.8. Biochemical Markers

**Figure 5:**
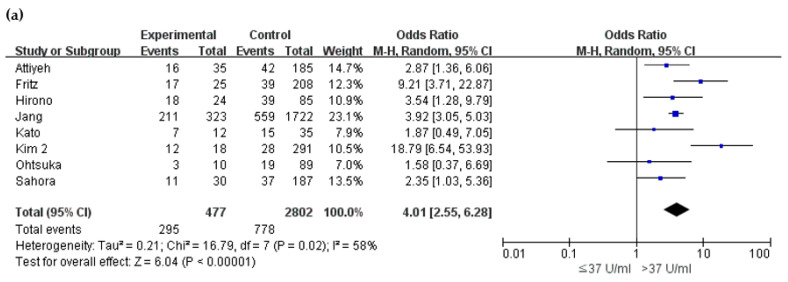

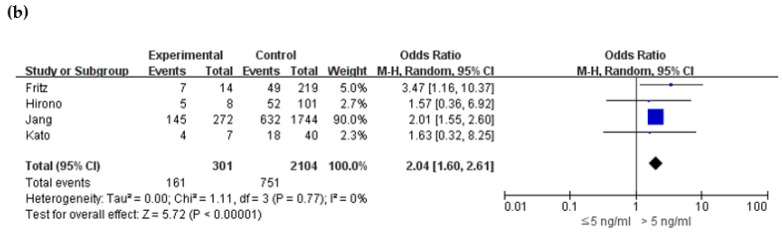
Forest plot demonstrates the incidence of malignancy in BD-IPMN in relation to biochemical markers. (**a**) carbohydrate antigen 19-9 and (**b**) carcinoembryonic antigen.

Carbohydrate antigen (CA) 19-9 with a cut-off level of 37 U/mL was examined in eight studies [15,21,23,24,25,26,36,40]. Among 3279 pooled patients, 477 patients (14.5%) had elevated CA 19-9 levels, of which 61.8% had malignant BD-IPMN, whereas only 27.8% of the normal CA 19-9 patients showed malignancy. The OR was 4.01 (95% CI 2.55–6.28, *p* < 0.001) (Table 2 and Figure 5a).

There were four studies [21,23,24,25] with pooled cohort of 2405 patients that reported the presence of carcinoembryonic antigen (CEA) with a cut-off level of 5 ng/mL. The malignancy rate among patients with elevated CEA and normal CEA was 53.5% and 35.7%, respectively. The OR for elevated CEA was 2.04 (95% CI 1.60–2.61, *p* < 0.001) (Table 2, Figure 5b).

## 3. Discussion

This study revealed that the parameters of symptoms, size, cystic wall thickening, presence of mural nodule, change in main pancreatic duct caliber, lymphadenopathy, CA 19-9, and CEA were the predictive features of malignancy in BD-IPMN. On the other hand, multilocularity of cyst and multiple cysts were not malignancy predictors.

The findings are in accordance with most of the widely used guidelines. The AGA guideline utilizes size, dilated main pancreatic duct, solid component, and positive cytology to determine the treatment strategy [6]. Reference size ≥ 3 cm, dilated main pancreatic duct, and associated solid component were considered risk factors, and presence of at least two of these would warrant endoscopic ultrasound-fine needle aspiration (EUS-FNA). In the case of positive cytology or presence of a solid component and a dilated pancreatic duct, surgery is indicated. Since this guideline is for asymptomatic neoplastic pancreatic cysts, the symptoms were not considered. 

Unlike the conservative AGA guidelines, the European study group proposes a more aggressive approach in BD-IPMN patients [7,8]. Presence of jaundice, positive cytology, enhancing mural nodule (≥5 mm), solid mass, and main pancreatic duct ≥10 mm are absolute indicators for surgery. Growth rate ≥5 mm/year, elevated serum CA 19-9 level, main pancreatic duct dilatation between 5–9.9 mm, cyst diameter ≥40 mm, new onset diabetes mellitus, acute pancreatitis, and enhancing mural nodule (<5 mm) are relative indicators wherein healthy patients may opt for surgery.

The IAP guidelines stratify the features into high-risk stigmata and worrisome features. The high-risk stigmata and worrisome features warrant surgery and EUS, respectively. High-risk stigmata include obstructive jaundice in a patient with cystic lesion of the head of the pancreas, enhancing mural nodule ≥5 mm, and main pancreatic duct ≥10 mm. Worrisome features include cyst ≥3 cm, enhancing mural nodule <5 mm, thickened/enhancing cyst walls, main duct size 5–9 mm, abrupt change in caliber of pancreatic duct with distal pancreatic atrophy, lymphadenopathy, increased CA 19-9 serum level, and cystic growth rate ≥5 mm/2 years. The features used by the IAP and European study group are similar. However, the IAP guidelines are slightly more conservative, wherein surgery is decided based on the EUS findings in patients with worrisome features.

In this study, all the features were included to validate those featuring in various guidelines. In addition, other features such as locularity, multiplicity, and CEA serum level were explored. The parameter of symptoms showed a significant association with malignancy. However, the symptoms could be heterogenous and often vague. They consisted of one or combinations of clinical findings such as abdominal pain, weight loss, pancreatitis, and jaundice. Therefore, it is difficult to define what symptom to look for and determine the appropriate treatment strategy. Notably, jaundice was found to be a significant predictor of malignancy by several studies [15,27,38,42]. In particular, a nomogram developed by Attiyeh et al. [15] automatically assigned a predicted probability of high-risk disease of “1” to patients with jaundice. Another symptom that showed high association with malignancy was weight loss. Among five studies that examined weight loss separately [15,19,20,33,42], all studies except one [33] found weight loss to be significantly associated with malignancy. While many symptoms depend on the patient’s report and tend to be subjective, jaundice and weight loss are symptoms that can be objectively quantified. Therefore, instead of considering symptoms as a whole, utilizing jaundice and weight loss to predict malignancy seemed reasonable, and studies defining the cut-off values for these symptoms should be warranted. Nevertheless, jaundice and weight loss are symptoms often associated with overt cancer and may have limited value in predicting earlier malignant transformation such as high-grade dysplasia. Our results showed that 27.4% of asymptomatic patients reported malignancy, demonstrating that absence of symptoms does not assure the absence of malignancy. Therefore, radiologic and biochemical changes may be more important in early detection of malignant transformations.

Previously, a cyst size of 3 cm was considered an absolute indication of BD-IPMN [10,51,52]. However, subsequent studies found that size alone was insufficient to predict malignancy, and although size correlated with malignancy risk, the safe cut-off limit was unclear [53,54,55,56]. The European study group does not consider a cyst size of 3 cm as an absolute indication, but rather considers the presence of other risk factors as determining factors, unless the diameter reaches 4 cm [7]. The IAP also stepped down the 3-cm size criteria from an absolute indication to a worrisome feature since the 2012 consensus guidelines [1,9]. Likewise, although the AGA states that size ≥3 cm increases the risk of malignancy by three times [57], size is not the sole determinant of the strategy [6]. In the present study, size ≥3 cm increased the malignancy risk by two times. Although size is a significant factor, its impact is not as great as that of other features. Hence, size alone has a limited potential in predicting malignancy.

Wall thickening is a feature considered exclusively in the guidelines by IAP. It was introduced in the 2012 consensus guideline [1]. In this study, wall thickening increased the malignancy risk by 2.5 times. However, it is uncertain whether the wall thickening was accompanied by enhancement in the studies. Other cystic characteristics such as multilocularity or multiplicity of cysts did not increase the risk of malignancy.

Mural nodule is one of the strongest and most consistent risk factors in all the guidelines. Mural nodule is an absolute indication according to the European study group, and it could be an indication if it is accompanied by main pancreatic duct dilatation >5 mm according to the AGA guidelines. The AGA found that solid component increased the risk by almost eight times after reviewing 816 patients in seven studies [57]. They found that the incidence of malignancy was 73% in patients with a mural nodule as compared to 23% in those without a mural nodule. In 25 studies with 4495 patients, the malignancy rate was 52.5% in those with a mural nodule and 20.4% in those without. Furthermore, this study found that the malignancy risk was four times higher in patients with mural nodule. Nevertheless, mural nodule is one of the highly predictive factors of malignancy. Recently, enhancement and size of mural nodule have received attention, and these factors were applied to the IAP and European study group guidelines [1,8,58,59,60,61]. However, the diagnostic performances vary according to the imaging modality used, and meta-analysis cannot be conducted with the limited number of studies. Therefore, this study did not sub-analyze the mural nodule feature by size or enhancement, and future studies are needed to clarify the effect of these factors.

The main pancreatic duct change is another consistent risk factor of malignancy. The European study group, AGA, and IAP guidelines include main pancreatic duct dilatation. The AGA did not provide a definition of main duct dilation, whereas the European study group and IAP defined duct dilatation as dilatation >5 mm [1,6,7,8,9,57]. The IAP and European study group further stratified the risk level according to the extent of dilatation. Main duct dilation between 5–9 mm and >1 cm is considered as worrisome feature and high-risk stigmata by the IAP, or as a relative and absolute indication by the European study group. In contrast, the AGA requires that the solid component be accompanied by main duct dilatation for it to qualify as an indication. Interestingly, the AGA did not find a significant association between dilated pancreatic duct and malignancy (OR, 2.38, 95% CI 0.71–8.00), but included it in their guidelines because the review was performed with resected IPMNs [6,57]. Nevertheless, the main duct dilatation is a well-recognized risk factor that was also confirmed in this meta-analysis. However, the reference cut-off values vary according to studies, and each guideline weighs the same criteria differently. Future efforts are required to reach a consensus. Another change often studied and considered in the IAP guidelines is the abrupt change in caliber. Although this may overlap with main duct dilatation and may be considered an extreme form of dilatation, its OR was the second highest in this study at 7.41. However, this was based only on four studies, and the true predictive value needs further validation.

Lymphadenopathy was recently added to the IAP guidelines during the 2017 revision [9]. There are no references to lymphadenopathy in the European study group or AGA guidelines. Although least attention was given to lymphadenopathy, it demonstrated the highest OR, showing 8.5 times increased risk of malignancy. There were only four studies with a pooled cohort of 390 patients, of which 6.2% had lymphadenopathy. More studies are needed to accurately evaluate the impact of lymphadenopathy in predicting the malignancy in BD-IPMN cases.

Finally, a biochemical marker, CA 19-9, was indicated as a relative risk factor in the European guidelines and as a worrisome feature in the revised 2017 IAP guidelines [7,9]. This study showed that elevated CA 19-9 above 37 U/mL had four times higher risk of malignancy, which is similar to the risk associated with mural nodule. In addition to CA 19-9, the role of CEA was examined, which posed twice the risk of malignancy when elevated above 5 ng/mL. However, only four studies were examined and its actual role needs to be further studied for a definitive conclusion.

There are several limitations in this study. First, all the studies included in this meta-analysis were observational studies, and potential biases are likely to be greater in such studies. Thus, the results should always be interpreted with caution. Second, the studies were conducted on resected IPMNs, thus limiting the knowledge regarding the natural course of the disease. Conversely, this ensures the most accurate pathologic diagnosis. Third, some features had slightly different or more specified definitions in the guidelines, e.g., for “enhanced” wall thickening, and different values for duct dilatations. However, for analysis with an adequate population, the features could not be too narrowly defined. Finally, as the risk of malignancy is likely to increase, an analysis of risk by combination of features or creating a predictive model would have been informative. 

## 4. Materials and Methods 

### 4.1. Literature Search Strategy

A literature search was conducted using the MEDLINE to identify a relevant study about the outcomes in patients with worrisome features or high-risk stigmata of IPMN and malignancy proven by surgery or biopsy. A combination of search terms, including IPMN, computed tomography (CT), magnetic resonance image (MRI), EUS, malignancy, worrisome features, or predictive features, were used. 

### 4.2. Inclusion/Exclusion Criteria

Studies were included if they met the following criteria: written in English, full-article, publication year between January 2000 and May 2019, patient with BD-IPMN diagnosed by CT, MRI or EUS and final pathological diagnosis by surgical resection or biopsy, and >10 patients in the study. We excluded case reports, case series with small sample size (<10 patients), review articles, editorials, consensus proceedings, studies without pathological diagnosis, not within field of interest, and insufficient or overlapping data.

### 4.3. Data Extraction and Quality Assessment

Two reviewers (W.K. and Y.H.) independently extracted the data from each study and resolved their disagreements by discussion or by consulting a third reviewer (J-Y.J.). The following data were collected from the studies that met the criteria. (1) Study—publication year, study design, and study location. (2) Cases—total number of BD-IPMN patients, frequency of pathologic malignancy in BD-IPMN, age, and sex. (3) Cystic morphology—maximum cyst size, presence of mural nodules, and maximum diameter of main pancreatic duct. (4) Clinical data—symptoms (jaundice, diabetes, pain, and weight loss), imaging methods, CA 19-9 level (normal value 0–37 U/mL), and CEA level (normal value 0–5 ng/mL). (5) Outcomes—cytology result and pathology result.

Malignant BD-IPMN was identified when there was histological evidence of BD-IPMN with invasive carcinoma or high-grade dysplasia after surgical resection, and cytological/histological evidence of high-grade dysplasia/malignant cells was found after FNA/biopsy of BD-IPMN with or without associated radiological signs of malignancy. 

The choice of the articles included in this review were in accordance with the Preferred Reporting Items for Systematic Reviews and Meta-Analyses statement (PRISMA) [62], and a PRISMA flowchart was formulated (Figure 1) for transparency of the conclusions reached by the authors. The quality of included studies was assessed using the Newcastle Ottawa Scale [63] by two reviewers (W.K. and Y.H.).

### 4.4. Data Analysis 

Interpretative analysis of the OR between positive and negative worrisome features in IPMN patients was performed. The OR of BD-IPMN with or without worrisome features/high-risk stigmata was calculated by dividing the total number of events by the total number of patients. If these specific data were not provided in a study, it was calculated by adding or subtracting the number of patients who had confirmed pathology and imaging data. The corresponding 95% CIs were calculated using exact methods. A meta-analysis of all eligible studies identified was then planned with the Review Manager software (RevMan) (version 5.3; The Cochrane Collaboration, The Nordic Cochrane Center, Copenhagen, Denmark) using a random-effects model. This model was used because we believe that the relevant variation in the risk is most likely a consequence of inter-study differences. Statistical analysis was performed for all stages of this meta-analysis in accordance with the MOOSE guidelines [64]. The quantity of heterogeneity and publication bias was assessed. A *p*-value < 0.050 was accepted as statistically significant. 

## 5. Conclusions

This study examined the parameters used to predict malignancy as specified by the most commonly used guidelines. This not only included clinical and radiographic features, but also biochemical features. The results confirmed that all the currently used features are valid. However, each guideline utilizes certain features and weighs the impact of each feature differently, resulting in different treatment strategies in BD-IPMN patients presenting similar features. This study hopes to contribute in making future guidelines more compatible and standardized.

## Figures and Tables

**Table 1:** Characteristics of included studies.

Study	Year	Study Period	No. of Patients	Mean Age (Years)	Male/Female	Type of IPMN	Benign	Malignant	Malignancy Proportion (%)	Diagnostic Modality
Akahoshi et al. [11]	2018	2006–2017	50	68	33/17	BD	33	17	34.0%	CT, MRI
Akita et al. [12]	2011	1992–2007	32	62.6	19/13	BD	20	12	37.5%	CT, MRI, MRCP
Arikawa et al. [13]	2011	2003–2008	25	65.2	20/5	BD	17	8	32.0%	CT, MRCP, EUS
Aso et al. [14]	2014	2006–2013	70	N/A	N/A	BD	42	28	40.0%	CT, MRCP, EUS
Attiyeh et al. [15]	2018	2005–2015	381	67	160/221	BD	276	105	27.6%	Not stated
Bournet et al. [16]	2009	1988–2005	53	63.9	52/47	BD, mixed	29	24	24.2%	CT, MRCP, EUS, ERCP
Carbognin et al. [17]	2006	1995–2005	29	Benign 64.7 Malignant 62.2	17/12	BD, mixed	11	18	62.1%	CT, MRI, MRCP
Chiu et al. [18]	2006	1995–2005	40	N/A	N/A	BD, MD, mixed	30	10	25.0%	CT
Correa-Gallego et al. [19]	2013	1994–2010	123	68	50/73	BD	87	36	29.3%	Not stated
Dortch et al. [20]	2015	2002–2013	66	68	26/42	BD	54	12	18.2%	CT, MRCP, EUS, FNA
Fritz et al. [21]	2014	2004–2012	233	N/A	95/138	BD	177	56	24.0%	CT, MRI
Harima et al. [22]	2015	2009–2014	15	N/A	N/A	BD	1	14	93.3%	CT, EUS
Hirono et al. [23]	2017	1999–2015	109	N/A	46/63	BD	52	57	52.3%	CT, EUS
Jang et al. [24]	2017	1992–2012	2258	65.0	1408/850	BD	1429	829	36.7%	CT, EUS
Kato et al. [25]	2015	1994–2012	47	66.2	30/17	BD	25	22	46.8%	Not stated
Kim YI et al. [26]	2015	1997–2013	324	62	179/145	BD	282	42	13.0%	CT, MRCP, EUS, ERCP
Kim TH et al. [27]	2015	2004–2012	177	63	108/69	BD	138	39	22.0%	CT, EUS
Koshita et al. [28]	2017	2005–2014	28	62.2	17/11	BD	14	14	50.0%	CT, MRCP, EUS, ERCP
Lee et al. [29]	2014	2002–2011	84	64.7	55/29	BD	68	16	19.0%	EUS
Maguchi et al. [30]	2011	N/A	29	N/A	N/A	BD	20	9	31.0%	CT, EUS
Mimura et al. [31]	2010	1998–2009	43	Benign 66.0 Malignant 66.7	29/14	BD, mixed	23	20	46.5%	CT, EUS
Nagai et al. [32]	2009	1984–2007	84	63	48/36	BD	47	37	44.0%	CT, ERCP, MRI, EUS
Nguyen et al. [33]	2015	1996–2012	66	69	26/42	BD	51	15	22.7%	CT, MRI, EUS
Ogawa et al. [34]	2008	2000–2006	49	64.9	39/20	BD	22	27	55.1%	CT
Ohno et al. [35]	2012	2001–2009	30	65.1	15/15	BD	19	11	63.3%	CT, ERCP, CE-EUS
Ohtsuka et al. [36]	2012	1990–2009	99	NA	60/39	BD	77	22	22.2%	CT, MRCP, US, EUS
Ridtitid et al. [37]	2016	2001–2013	135	65.2	71/64	BD	117	18	13.3%	CT, MRI, EUS
Robles et al. [38]	2016	2006–2014	120	57.9	65/55	BD	84	36	30.0%	CT, MRI, EUS
Rodriguez et al. [39]	2007	1990–2005	145	67*	62/83	BD	113	32	22.1%	CEUS, CT, MRI
Sahora et al. [40]	2013	1995–2012	217	N/A	82/135	BD, mixed	169	48	22.1%	CT, MRI, MRCP, EUS
Salvia et al. [41]	2007	2000–2003	20	58	10/10	BD	18	2	10.0%	US, MRI, MRCP, CEUS, EUS, ERCP
Schmidt et al. [42]	2007	1991–2006	103	63	50/53	BD	83	20	19.4%	CT, MRI, ERCP, EUS
Seo et al. [43]	2016	2011–2013	60	64.3	35/25	BD	52	8	13.3%	CT, MRI
Serikawa et al. [44]	2006	1992–2005	56	65.8	42/14	BD	49	7	10.3%	US, EUS, CT, ERCP, MRCP
Shimizu et al. [45]	2020	1996–2014	466	67.9	274/192	BD, MD, mixed	208	258	55.4%	CT, EUS, MRCP
Strauss et al. [46]	2016	2004–2012	168	N/A	N/A	BD	126	42	25.0%	CT, MRI, MRCP
Takeshita et al. [47]	2008	2002–2006	46	65	28/25	BD	38	8	17.4%	CT
Tang et al. [48]	2008	1995–2006	31	66.5	10/21	BD	26	5	16.1%	CT, MRI, MRCP, ERCP, EUS
Wong et al. [49]	2013	2000–2010	105	68	47/58	BD	43	62	59.0%	CT, MRI, EUS
Woo et al. [50]	2009	1998–2005	85	63	50/35	BD	71	14	16.5%	CT, EUS, ERCP, MR

N/A, not available; IPMN, intraductal papillary mucinous neoplasm; BD, branch duct; MD, main duct; CT. computed tomography; MRI, magnetic resonance image; MRCP, magnetic resonance cholangiopancreatography; ERCP, endoscopic retrograde cholangiopancreatography; US, ultrasonography; CEUS, contrast-enhanced ultrasonography; EUS, endoscopic ultrasonography; CE-EUS, contrast-enhanced endoscopic ultrasonography; FNA, fine needle aspiration. * in median.

**Table 2:** Summary of clinical, radiographic, and biochemical parameters.

Parameters	No. Studies	No. of Patient	No. of Positive Feature (%)	No. of Malignancy (%)	No. of Malignancy among Positive Features (%)	No. of Malignancy among Negative Features (%)	OR	95% CI	*p*-Value
Symptoms (+)	16	2844	1089 (38.3)	966 (34.0)	369 (33.9)	597 (34.0)	1.35	1.01, 1.79	0.040
Cyst size (≥3 cm)	22	4446	2091 (47.0)	1414 (31.8)	814 (38.9)	605 (25.7)	1.90	1.51, 2.40	<0.001
Wall thickening	9	689	105 (15.2)	192 (27.9)	54 (51.4)	138 (23.6)	2.53	1.50, 4.27	<0.001
Multilocular	7	741	389 (52.5)	183 (24.7)	105 (27.0)	78 (22.2)	0.92	0.63, 1.35	0.68
Multiplicity	8	1058	350 (33.1)	272 (25.7)	84 (24.0)	188 (26.6)	0.76	0.55, 1.04	0.09
Mural nodule	25	4495	1610 (35.8)	1434 (31.9)	845 (52.5)	589 (20.4)	4.10	3.38, 4.97	<0.001
MPD dilatation	15	3499	1482 (42.4)	1190 (34.0)	698 (47.1)	492 (24.4)	2.98	2.11, 4.21	<0.001
> 5 mm	8	3098	1305 (42.1)	1031 (33.3)	607 (46.5)	424 (23.6)	2.85	1.90, 4.26	<0.001
> 6 mm	5	270	107 (39.6)	125 (46.3)	70 (65.4)	55 (33.7)	3.86	1.63, 9.11	0.002
> 7 mm	2	131	70 (53.4)	72 (55.0)	21 (30.0)	13 (21.3)	2.69	0.42, 17.16	0.29
Abrupt caliber change	4	467	34 (7.3)	74 (15.8)	18 (52.9)	56 (12.9)	7.41	2.49, 22.06	<0.001
Lymphadenopathy	4	390	70 (17.9)	70 (17.9)	14 (20.0)	56 (15.3)	8.55	3.25, 22.51	<0.001
CA 19-9 (> 37 U/mL)	8	3279	477 (14.5)	1073 (32.7)	295 (61.8)	778 (27.8)	4.01	2.55, 6.28	<0.001
CEA (> 5 ng/mL)	4	2405	301 (12.5)	912 (37.9)	161 (53.5)	751 (35.7)	2.04	1.60, 2.61	<0.001

OR, odd ratio; CI, confidence interval; MPD, main pancreatic duct; CA, carbohydrate antigen; CEA, carcinoembryonic antigen.
